# Psychosis detection in dementia: a systematic review of diagnostic test accuracy studies

**DOI:** 10.3389/frdem.2024.1377191

**Published:** 2024-06-13

**Authors:** Kayla Atchison, Pauline Wu, Jennifer A. Watt, Dallas Seitz, Zahinoor Ismail, Zahra Goodarzi

**Affiliations:** ^1^Department of Medicine, University of Calgary, Calgary, AB, Canada; ^2^Department of Medicine, University of Toronto, Toronto, ON, Canada; ^3^Department of Psychiatry, University of Calgary, Calgary, AB, Canada; ^4^Hotchkiss Brain Institute, University of Calgary, Calgary, AB, Canada; ^5^O'Brien Institute for Public Health, University of Calgary, Calgary, AB, Canada

**Keywords:** psychosis, hallucinations, delusions, dementia, detection, sensitivity, systematic review

## Abstract

**Introduction:**

Symptoms of psychosis, characterized by delusions and hallucinations, are commonly experienced by persons living with dementia. A systematic review was completed to identify tools to evaluate symptoms of psychosis compared to a reference standard in persons with dementia. Articles reporting correlation values between psychosis tools were also identified.

**Methods:**

The search concepts psychosis, dementia, and diagnostic accuracy were used to search MEDLINE, PsycINFO, and Embase. Included articles meeting the primary objective described a tool to assess symptoms of psychosis, delusions, or hallucinations in persons with dementia, a reference standard form of diagnostic assessment for psychosis, and diagnostic accuracy outcomes for the psychosis tool. Secondary objective articles reported correlation values between two or more psychosis tools in persons with dementia.

**Results:**

One study met the primary objective and described the sensitivity and specificity of the Neuropsychiatric Inventory (NPI) and Columbia University Scale for Psychopathology in Alzheimer's Disease (CUSPAD) in identifying symptoms of psychosis, hallucinations, and delusions. The sensitivity of the NPI and CUSPAD in identifying symptoms of psychosis was 83 and 90%, respectively. Nine studies meeting the secondary objective described eleven unique tools and examined the degree to which tools used to assess psychotic symptoms in persons with dementia were related.

**Discussion:**

The NPI and CUSPAD were identified in a single study as psychosis tools that have been evaluated against a reference standard of psychosis assessment in persons with dementia. Various tools to assess the burden of psychotic symptoms in persons with dementia exist, but the diagnostic accuracy of existing tools remains understudied. Further research on the comparative utility and diagnostic accuracy is required for all psychosis tools used with persons with dementia.

## 1 Introduction

Psychosis, characterized by delusions and hallucinations, is part of a clinically relevant group of symptoms referred to as behavioural and psychological symptoms of dementia (BPSD) (Rayner et al., 2006). Between 34 and 63% of persons with dementia are estimated to experience psychotic symptoms at some point in their illness (Pessoa et al., 2023). Psychosis is associated with faster cognitive decline, higher caregiver burden, and greater risk of long-term care placement (Ropacki and Jeste, 2005; Allegri et al., 2006; Toot et al., 2017; Connors et al., 2018; Walsh et al., 2021). Psychosis, among other BPSD, creates significant challenges for the person experiencing dementia and their caregivers. There is a need for early detection and treatment initiation for psychotic symptoms in dementia to reduce the symptom burden on patients and caregivers (Fischer et al., 2020; Ismail et al., 2022). Psychosis in persons without dementia differs from those with dementia given the specific pathology and neurodegeneration associated with dementia syndromes can lead to varied underlying causes and presentations as well as interventions to address symptoms differ (Sabbagh et al., 2023).

Understanding how to best assess symptoms of psychosis in dementia is complicated by inconsistencies in the terminology used in the literature and limited by the research gap in psychosis assessment tools focused on dementia populations (Seiler et al., 2020). Commonly used tools for the assessment of symptoms of psychosis in the context of dementia include the Columbia University Scale for Psychopathology in Alzheimer's Disease (CUSPAD), Behavioral Pathology in Alzheimer's Disease (BEHAVE-AD) rating scale, Neuropsychiatric Inventory (NPI), NPI-Nursing Home version (NPI-NH), and Consortium to Establish a Registry for Alzheimer's Disease Behavior Rating Scale for Dementia (CERAD-BRSD) (Cohen-Mansfield and Golander, 2011; Cerejeira et al., 2012). Current measures used to evaluate the burden of psychotic symptoms in persons with dementia may be limited by the reliance on caregiver observational reports that may introduce bias due to lack of awareness of psychotic symptoms as well as patient interview-based approaches that may introduce bias due to lack of insight, or tools looking at many symptoms of BPSD and not focused on psychosis. Observational tools allow for psychosis assessment in persons with more severe cognitive issues, or difficulty with communication. Psychosis differs from primary symptoms of dementia in that the focus is on the presence of delusions and hallucinations—unlike other cognitive symptoms (Sabbagh et al., 2023). Diagnostic criteria like the International Psychogeriatric Association (IPA) criteria (Cummings et al., 2020) and the Alzheimer's Association International Society to Advance Alzheimer's Research and Treatment (ISTAART-AA) criteria (Fischer et al., 2020) specifically address psychosis in the context of dementia. The IPA criteria define psychosis in major and mild neurocognitive disorder while the ISTAART research criteria support the classification of psychosis in Alzheimer's disease, irrespective of cognitive and functional status (Cummings et al., 2020; Fischer et al., 2020).

Existing studies have identified commonly used tools to assess symptoms of psychosis in dementia but have not evaluated the diagnostic accuracy of psychosis tools against established reference standards. Diagnostic accuracy studies reporting sensitivity, specificity, and likelihood ratios are crucial to understand which tools to use in practice. To date, no systematic reviews of diagnostic accuracy for psychosis tools in dementia have been completed. There is a need for valid, objective tools to assess the presence, severity, and frequency of psychotic symptoms in persons with dementia to accurately inform treatment. It is unclear what the diagnostic accuracy (e.g., sensitivity, specificity) is of existing psychosis tools compared to a reference standard form of psychosis assessment [i.e., clinical interview, Diagnostic and Statistical Manual of Mental Disorders (DSM) criteria (American Psychiatric Association, 2013), International Classification of Diseases (ICD) criteria (World Health Organization, 1993), IPA criteria (Cummings et al., 2020), or ISTAART-AA criteria (Fischer et al., 2020)].

A systematic review was completed to identify diagnostic accuracy studies of tools that evaluate symptoms of psychosis compared to a reference standard in persons with dementia. The secondary objective of the systematic review was to identify articles that evaluated the convergent validity, concurrent validity, or correlation between two or more psychosis tools.

## 2 Methods

The systematic review has been reported according to the Preferred Reporting Items for a Systematic Review of Diagnostic Test Accuracy Studies Checklist (McInnes et al., 2018).

### 2.1 Search strategy

Three electronic databases were searched (MEDLINE, PsycINFO, Embase) on July 20, 2023, using the search concepts *dementia, psychosis*, and *diagnostic accuracy*. A fourth search concept with specific diagnostic accuracy key words was included to narrow the focus of the search. The database search strategy used has been reported (Supplementary Table 1). The search excluded literature related to schizophrenia or bipolar disorder. The reference lists of included articles and references of review articles meeting title and abstract inclusion criteria were hand-searched. Search strategy key words were used to search grey literature sources identified using the Grey Matters Tool Lite (Canadian Agency for Drugs and Technologies in Health, 2018). The first 200 results from each grey literature source were screened (Supplementary Table 2). No date, publication status, or language limits were applied. Non-English language articles were translated into English using Google Translate.

### 2.2 Inclusion criteria

At the stage of title and abstract review, articles had to include a tool used to measure symptoms of psychosis in a dementia population. Dementia populations included persons with any type of dementia or major neurocognitive disorders. Literature focused on populations with Huntington's disease, Parkinson's disease, and mild or subjective cognitive impairment were excluded. Psychosis tools at the title and abstract review stage could be specific to psychosis or global measures of neuropsychiatric symptoms. At the stage of full text review, studies meeting inclusion criteria for the primary objective had to include a tool used to assess symptoms of psychosis, delusions, or hallucinations in persons with dementia, a reference standard form of diagnostic assessment for psychosis (i.e., clinical interview, DSM criteria, ICD criteria, IPA criteria, ISTAART criteria), and diagnostic accuracy outcomes for the psychosis tool (e.g., sensitivity, specificity). Diagnostic accuracy outcomes had to be reported specifically for the dementia population.

Articles meeting inclusion criteria for the secondary objective were those excluded at the level of full text in primary objective screening based on not reporting diagnostic accuracy outcomes (study used a psychosis tool and form of reference assessment but no data were provided to produce diagnostic accuracy estimates) or not using a reference assessment (study reported using two or more tools to identify psychosis in persons with dementia, however, no tools used were considered reference standard methods of assessment). Articles that reported correlation values between two or more psychosis tools in populations with dementia were included as meeting the secondary review objective.

### 2.3 Data extraction

Participant demographic information, dementia type and severity, symptoms of psychosis reported (e.g., hallucinations, delusions), psychosis prevalence, and information on the psychosis tools(s) were extracted from all included articles. Information on the reference standard assessment and the per-patient diagnostic accuracy outcomes (sensitivity, specificity, positive and negative predictive values, and positive and negative likelihood ratios) were extracted from articles informing the primary objective while correlation coefficients (Spearman's Rank Correlation Coefficient, Pearson's Correlation Coefficient) and related *p*-values were extracted from articles meeting the secondary objective.

### 2.4 Risk of bias

The Quality Assessment of Diagnostic Accuracy Studies (QUADAS)-2 (Whiting et al., 2011) tool was used to assess the risk of bias and concerns regarding applicability to the review question in studies meeting the primary objective. Study quality for articles meeting the secondary objective was assessed using the COnsensus-based Standards for the selection of health Measurement INstruments (COSMIN) checklist (Mokkink et al., 2018). Two reviewers independently screened, extracted, and completed quality assessments for articles. Conflicts in title and abstract screening, extraction, and quality assessment were resolved among reviewers while a third reviewer resolved conflicts at the level of full text screening.

### 2.5 Synthesis

Characteristics of study participants, index tools identified, and reference assessments used were descriptively synthesized. Diagnostic accuracy outcomes for the primary objective were reported and correlational values for the secondary objective were tabulated. Diagnostic test accuracy and correlation outcomes were reported separately for symptoms of psychosis, hallucinations, and delusions where possible. A quantitative synthesis of the prevalence of psychosis or diagnostic accuracy outcomes was not possible given the limited number of studies identified.

## 3 Results

**Figure 1:**
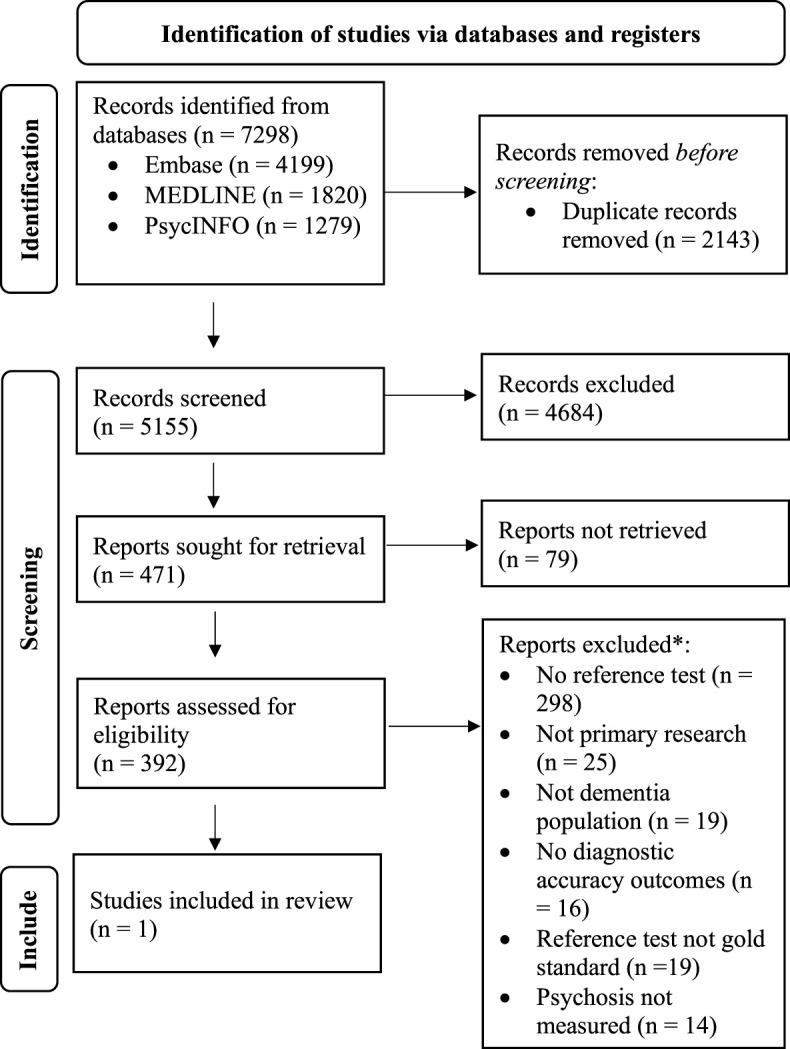
PRISMA flow diagram of study inclusion in systematic review (Page et al., 2021). Ten studies excluded based on *Reference test not gold standard* or *No diagnostic accuracy outcomes* are reported on in the present systematic review based on the correlation values or rates of clinical improvement they report between psychosis index tools.

Five thousand one hundred fifty-five articles were returned from database searches after duplicates were removed and 471 articles were reviewed in full text (Figure 1). Following full text review, one study met the inclusion criteria for the primary objective. Nine studies originally excluded based on not reporting diagnostic accuracy outcomes or not using a reference standard form of assessment, met the criteria for the secondary objective. One additional article discussing clinical improvement in psychotic symptoms based on a psychosis tool was included in the review for descriptive purposes. Reviewers disagreed on 6.05% of articles at the stage of title and abstract review and 1.79% at the stage of full text review.

### 3.1 Studies evaluating psychosis tool against reference standard (*n* = 1)

The single included study (Rapoport et al., 2001) was completed in Canada in 2001 with persons with mild to moderate dementia recruited from memory disorder clinics (Table 1). Of the 61 participants enrolled, the majority were married (72.1%) and female (55.74%). Participants had a mean age of 74.4 and were diagnosed with probable Alzheimer's disease, probable or possible vascular dementia, or dementia with Lewy bodies.

**Table 1:** Study and participant characteristics of included study evaluating an index psychosis tool compared to a reference standard.

**References, Country**	**Age [mean (SD)]**	***n***	**% female**	Prevalence of psychosis (%)^*^	**MMSE score [mean (SD)]**	**Dementia type**	**Recruitment setting; evaluation location**	**Index tool(s)**	**Reference standard**	**Is the index tool self- or clinician-rated?**
Rapoport et al. (2001), Canada	74.4 (9.13)	61 (not all participants completed each index test)	55.74%	• NPI [psychosis (39%), delusions (33.9%), hallucinations (12.1%)] • CUSPAD [psychosis (40.8%), delusions (36.7%), hallucinations (10.4%)]	Mild to moderate dementia (MMSE NR)	Probable AD (44.26%), DLB (9.84%), Vascular dementia (49.18%)	Memory disorders clinic; NR	• NPI (psychosis, delusions, hallucinations) • CUSPAD (psychosis, delusions, hallucinations)	Clinical interview	Caregiver report

^*^Prevalence calculations from clinical interview for each domain of each index tool based on the total number of participants that completed the respective index test assessment.

AD, Alzheimer's disease; CUSPAD, Columbia University Scale for Psychopathology in Alzheimer's disease; DLB, dementia with Lewy bodies; MMSE, Mini-Mental State Examination; *n*, total number of participants; NPI, Neuropsychiatric Inventory; NR, not reported; SD, standard deviation.

The NPI and CUSPAD (index psychosis tools) were compared to clinical interview findings (reference standard psychosis assessment) to identify delusions, hallucinations, and psychosis in participants. The psychiatric clinical interview took place with the patient and caregiver. The presence of delusions or hallucinations, extracted from the patient record following the clinical interview, were dichotomously coded as yes or no. For the participants completing the NPI, the prevalence of psychosis, delusions, and hallucinations according to the clinical interview was 39% (*n* = 23/59), 33.9% (*n* = 20/59), and 12.1% (*n* = 7/58), respectively. For the participants completing the CUSPAD, the prevalence of psychosis, delusions, and hallucinations according to the clinical interview was 40.8% (*n* = 20/49), 36.7% (*n* = 18/49), and 10.4% (*n* = 5/48), respectively. Single sensitivity (SN) and specificity (SP) values for symptoms of psychosis (NPI: SN = 82.6%, SP = 91.7%; CUSPAD: SN = 90%, SP = 89.7%), delusions (NPI: SN = 85%, SP = 92.3%; CUSPAD: SN = 77.8%, SP = 87.1%), and hallucinations (NPI: SN = 57.1%, SP = 92.2%; CUSPAD: SN = 80%, SP = 90.7%) were reported for each tool. The tool cut-off and the area under the curve were not reported (Table 2).

**Table 2:** Diagnostic accuracy values from included study evaluating an index tool compared to a reference standard.

**References, Country**	**Index test**	**Cut-off**	**SN**	**SP**	**PPV**	**NPV**	**LR(+)**	**LR(–)**	**AUC**	**Optimal cut-off**
Rapoport et al. (2001), Canada	NPI-psychosis	NR	0.826	0.917	0.864	0.892	9.952	0.190	NR	NR
	NPI-delusions	NR	0.850	0.923	0.850	0.923	11.039	0.163	NR	NR
	NPI-hallucinations	NR	0.571	0.922	0.500	0.940	7.321	0.465	NR	NR
	CUSPAD-psychosis	NR	0.900	0.897	0.857	0.929	8.738	0.111	NR	NR
	CUSPAD-delusions	NR	0.778	0.871	0.778	0.871	6.031	0.255	NR	NR
	CUSPAD-hallucinations	NR	0.800	0.907	0.500	0.975	8.602	0.221	NR	NR

AUC, area under the curve; CUSPAD, Columbia University Scale for Psychopathology in Alzheimer's disease; LR(–), negative likelihood ratio; LR(+), positive likelihood ratio; NPV, negative predictive value; NPI, Neuropsychiatric Inventory; NR, not reported; PPV, positive predictive value; SN, sensitivity; SP, specificity.

Bias in the included study resulted from not pre-specifying a cut-off value, unclear reporting on if the index test examiner was blind to the reference standard assessment results, lack of blinding of the reference standard assessor to the results of the index tests, unclear reporting on the time between index and reference standard assessments, and not including all enrolled participants in the analysis (Table 3). The included study had no concerns related to applicability to the review question.

**Table 3:** Risk of bias of included study evaluating an index tool compared to a reference standard assessed using QUADAS-2 tool.

**References, Country**	**Domain 1**		**Domain 2**		**Domain 3**		**Domain 4**
	**Could selection of patients have introduced bias?**	**Is there concern that the included patients do not match the review question?**	**Could the conduct or interpretation of the index test have introduced bias?**	**Is there concern that the index test, its conduct, or interpretation differ from the review question?**	**Could the ref standard, its conduct, or its interpretation have introduced bias?**	**Is there concern that the target condition as defined by the ref standard does not match the review question?**	**Could the patient flow have introduced bias?**
Rapoport et al. (2001), Canada	Low	Low	High	Low	High	Low	High

### 3.2 Studies evaluating convergent validity, concurrent validity, or correlation between psychosis tools (*n* = 9)

Nine studies examining the correlational values between psychosis tool scores were identified. Studies included between 40 and 405 persons with dementia, were comprised of between 43.79 and 76.71% female participants, and had mean ages ranging from 69.36 to 85.45 (Supplementary Table 3). The most common dementia diagnosis in study samples was Alzheimer's disease. The reported mean Mini-Mental State Examination (MMSE) scores were between 8.99 and 19.20. Participants were recruited from nursing homes, assisted living facilities, residential hospitals, a cognitive and behavioral disorders unit, outpatient dementia clinics, and a cognitive neuroscience clinic.

Risk of bias in correlational studies resulting in doubtful ratings for reliability assessments was from lack of reporting of the time interval between assessments and from lack of reporting or calculation of an intra-class correlation coefficient (Supplementary Table 4). Studies reporting on criterion or construct validity did not have quality concerns related to these domains.

Eleven unique tools were identified including the NPI (Cummings et al., 1994; Silveri et al., 2004; de Medeiros et al., 2010) (*n* = 3/9), NPI-Korean version (Youn et al., 2008) (*n* = 1/9), and NPI-Spanish version (Boada et al., 2002) (*n* = 1/9); NPI-Questionnaire (NPI-Q) (Hu et al., 2023) (*n* = 1/9) and NPI-Q-Spanish version (Boada et al., 2002, 2005) (*n* = 2/9); NPI-NH (Cohen-Mansfield and Golander, 2011) (*n* = 1/9) and NPI-NH-Spanish version (Boada et al., 2005) (*n* = 1/9); NPI-Clinician Rating Scale (NPI-C) (de Medeiros et al., 2010; Stella et al., 2013) (*n* = 2/9); BEHAVE-AD (Cummings et al., 1994; Cohen-Mansfield and Golander, 2011) (*n* = 2/9); Brief Psychiatric Rating Scale (BPRS) (de Medeiros et al., 2010; Stella et al., 2013) (*n* = 2/9); Behavior Rating Scale for Dementia-Short Form (BRSD-SF) (Cohen-Mansfield and Golander, 2011) (*n* = 1/9); BRSD-Korean version (BRSD-K) (Youn et al., 2008) (*n* = 1/9); CUSPAD (Cohen-Mansfield and Golander, 2011) (*n* = 1/9); Mild Behavioral Impairment Checklist (MBI-C) (Hu et al., 2023) (*n* = 1/9); and Survey Psychiatric Assessment Schedule (SPAS) (Silveri et al., 2004) (*n* = 1/9). Tools used patient report, caregiver report, professional caregiver report, or were clinician-rated (Table 4).

**Table 4:** Description of all tools identified in studies meeting primary and secondary review objectives.

**Index psychosis tool**	**Tool description**	**Information source**	**Time to complete**	**Number of items**	**Copyright restrictions**
NPI (Cummings et al., 1994)	Assesses neuropsychiatric symptom frequency and severity in dementia as well as related caregiver distress across 12 domains	Caregiver (preferably living with patient)	15 min (entire tool)	• Delusions−9 items • Hallucinations−7 items	Copyright
NPI-NH (Wood et al., 2000)	Characterizes neuropsychiatric symptom frequency and severity across 12 symptom domains as well as occupational disruptiveness. For use in nursing homes	Professional caregivers	Unclear	• Delusions−6 items • Hallucinations−7 items	Copyright
NPI-Q (Kaufer et al., 2000)	Provides neuropsychiatric symptom severity and distress ratings across 12 symptom domains. Brief assessment in clinical practice settings	Caregivers, reviewed for completeness by clinician	5 min or less (entire tool)	• Delusions−1 item • Hallucinations−1 item	Copyright
NPI-C (de Medeiros et al., 2010)	Assesses occurrence, severity, and meaningful change in neuropsychiatric symptoms across 14 symptom domains. For use in various settings and trial types	Clinician-rated, incorporates information from patients and caregivers as well as clinical judgement	Unclear	• Delusions−8 items • Hallucinations−7 items	Copyright
CUSPAD (Devanand et al., 1992)	Assesses psychosis, behavioral disturbances, and depression in persons with Alzheimer's disease across six subscales	Trained interviewer administers tool to caregiver informant	10–25 min (entire tool)	• Delusions−11 items • Hallucinations−5 items	None
BEHAVE-AD (Sclan et al., 1996)	Assesses behavioral symptoms in Alzheimer's disease across seven symptom clusters	Caregiver and other informants	20 min (entire tool)	• Paranoid and Delusional Ideation−7 items • Hallucinations−5 items	Copyright
BRSD-SF (Tariot et al., 1995)	Rates psychopathology in probable Alzheimer's Disease across seven subscales	Trained interviewer administers tool to caregiver informant and makes judgment on validity of caregiver's response	Unclear	• Delusions−2 items • Hallucinations−1 item	Unclear
BRSD-Korean (Youn et al., 2008)	Rates psychopathology in probable Alzheimer's Disease across six subscales	Trained interviewer administers tool to caregiver informant and makes judgment on validity of caregiver's response	Unclear	Psychotic symptoms−5 items	Unclear
BPRS (Ventura et al., 1993)	Assesses the level of 18 symptom constructs in persons with psychotic symptoms	Clinician-rated	Unclear	• Delusions−1 item • Hallucinations−1 item	None
MBI-C (Ismail et al., 2017)	Assesses mild behavioral impairment symptoms across five domains	Patient, close informant, or clinician	7–10 min (entire tool), 1–2 min (psychosis domain)	Psychosis−5 items	Free for non-commercial use
SPAS (Bond et al., 1980)	Examines mental state in older adults across three sections	Patient report	Unclear	Psychotic symptoms−10 items	Unclear

BEHAVE-AD, Behavioral Pathology in Alzheimer's Disease Rating Scale; BPRS, Brief Psychiatric Rating Scale; BRSD-SF, Behavior Rating Scale for Dementia-Short Form; BRSD-Korean, Behavior Rating Scale for Dementia-Korean Version; CUSPAD, Columbia University Scale for Psychopathology in Alzheimer's Disease; MBI-C, Mild Behavioral Impairment Checklist; min, minutes; NPI-C, Neuropsychiatric Inventory-Clinician Rating Scale; NPI-NH, Neuropsychiatric Inventory-Nursing Home; NPI, Neuropsychiatric Inventory; NPI-Q, Neuropsychiatric Inventory-Questionnaire; SPAS, Survey Psychiatric Assessment Schedule.

Seven studies compared two tools while one study each looked at three (de Medeiros et al., 2010) and four (Cohen-Mansfield and Golander, 2011) tools. Tools were used to assess symptoms of delusions or hallucinations independently, or psychosis as a cluster of symptoms. The correlation values between total scale scores or total symptom domain scores are discussed in text and reported in Supplementary Table 5. Correlation values between frequency or severity components of scale scores are reported in Supplementary Table 5.

The NPI was compared to the BPRS (psychosis: *r* = 0.56), BEHAVE-AD (delusions: *r* = 0.76; hallucinations: *r* = 0.74), and SPAS (not reported). The NPI-Korean version was compared to the BRSD-Korean version [delusions (NPI) and psychosis (BRSD-K): *r* = 0.39; hallucinations (NPI) and psychosis (BRSD-K): *r* = 0.93]. The NPI-NH was compared to the BEHAVE-AD (delusions: *r* = 0.75; hallucinations: *r* = 0.80), BRSD-SF (delusions: *r* = 0.53; hallucinations: *r* = 0.55), and CUSPAD (delusions: *r* = 0.82; hallucinations: *r* = 0.57). The NPI-Q was compared to the MBI-C (psychosis: *r* = 0.49) while the NPI-Q-Spanish version was compared to the NPI-Spanish version (delusions: *r* = 0.93; hallucinations: *r* = 0.91) and the NPI-NH-Spanish version (delusions: *r* = 0.37). The NPI-C was compared to the BPRS (psychosis: *r* = 0.60; delusions: *r* = 0.71; hallucinations: *r* = 0.43). The BEHAVE-AD was compared to the NPI (reported above), NPI-NH (reported above), BRSD-SF (delusions: *r* = 0.52; hallucination: *r* = 0.51), and CUSPAD (delusions: *r* = 0.58; hallucinations: *r* = 0.60).

### 3.3 Studies reporting clinical improvement in psychotic symptoms based on psychosis tool (*n* = 1)

A single study (Ismail et al., 2013) described the clinical improvement of psychosis in persons with dementia based on three psychosis tools [Empirical-BEHAVE-AD, Neurobehavioral Rating Scale (NBRS), NPI]. The NBRS detected the most cases of psychosis compared to the NPI and E-BEHAVE-AD. All three tools performed equally in detecting improvements in psychosis.

## 4 Discussion

The NPI and CUSPAD were identified as psychosis tools that have been evaluated against a reference standard of psychosis assessment in persons with dementia in a single study. Nine studies describing eleven unique tools examined the degree to which tools used to assess psychotic symptoms in persons with dementia were related. Various tools to assess the burden of psychotic symptoms in persons with dementia exist, but the diagnostic accuracy of existing tools remains understudied.

### 4.1 Diagnostic accuracy findings

The NPI and CUSPAD are tools that can be used to assess symptoms of psychosis in the context of dementia (Cohen-Mansfield and Golander, 2011). The CUSPAD had the highest sensitivity compared to the NPI when identifying psychosis (90%) or hallucinations (80%). The NPI had the highest sensitivity when identifying delusions (85%). Both the CUSPAD and NPI had adequate sensitivity to detect symptoms of psychosis and may be considered for use in practice. There is a need for more studies that compare psychosis tools to reference standard forms of psychosis assessment to better understand the diagnostic accuracy of the index psychosis detection tools.

### 4.2 Correlational findings

The NPI was the most studied tool in included studies with variations of the NPI (i.e., NPI-Q, NPI-NH, NPI-C) also commonly examined. The NPI seemed to best correlate with the NPI-Q for the assessment of delusions as well as the NPI-Q and BRSD-K (psychosis items) for the assessment of hallucinations. The NPI-NH seemed to be most aligned with the CUSPAD to assess delusions. The NPI-C had the strongest correlation with the BPRS for psychosis items and the BEHAVE-AD had a similar correlation to both the NPI and NPI-NH for the assessment of delusions and hallucinations.

Examining the strength of the correlation between existing psychosis tools supports a comparative understanding of the utility of each tool in clinical practice. There remains a limited understanding of the diagnostic accuracy of each tool in identifying psychosis in dementia and therefore the correlational findings may have limited clinical application. There were no instances where more than one study assessed the correlation between the same two tools or components of the same tools. Having only information on tool correlations from one study limits the interpretation of the correlational values between tools.

### 4.3 Psychosis tool utility and considerations

All tools except the MBI-C, SPAS, and BPRS were developed for assessing behavioral and/or psychological symptoms in dementia. The MBI-C was developed for persons with normal cognition or mild cognitive impairment, to identify neuropsychiatric symptoms meeting mild behavioral impairment criteria (Ismail et al., 2017), the SPAS for older adults (Bond et al., 1980), and the BPRS for persons with psychiatric symptoms (Ventura et al., 1993). The CUSPAD and BRSD assess psychopathology (Devanand et al., 1992; Tariot et al., 1995), the BEHAVE-AD assesses behavioral symptoms (Sclan et al., 1996), and the NPI and its variants are global assessments that look at a range of behavioral changes in persons with dementia but can also function to assess individual behavioral domains (Cummings et al., 1994; de Medeiros et al., 2010; Stella et al., 2013).

Tools that had more items were found to detect more symptoms (Cohen-Mansfield and Golander, 2011) therefore in practice, clinicians should consider the range of psychotic symptoms covered within the tool before use. Further research is required to understand the impact the environmental setting (e.g., nursing home, community) may have on existing measures.

The NPI, NPI-Q, CUSPAD, BEHAVE-AD, BRSD-SF, and BRSD-K rely on caregivers' reports of symptoms to complete the assessment. It was noted that differences may exist in patient experiences and caregiver perceptions of psychotic symptoms (Silveri et al., 2004). Further, informal caregivers have been found to rate the occurrence and severity of hallucinations and delusions higher than ratings of formal caregivers (Cohen-Mansfield et al., 2013). The BRSD-SF and BRSD-K stipulate that the trained interviewer makes a judgement on the validity of the caregivers' responses. The NPI-NH relies on information provided by professional caregivers while the NPI-C is a clinician-rated assessment taking into account patient and caregiver interview findings as well as clinical judgement (Stella et al., 2013).

The NPI differentiates between the severity and frequency of symptoms which may inform treatment selection (Cummings et al., 1994). The inclusion of caregiver distress in the NPI, NPI-Q, and NPI-C and occupational disruptiveness in the NPI-NH as part of the assessment may help inform the symptom burden relating to the patient as well as the caregiver (Cummings et al., 1994; Kaufer et al., 2000; Wood et al., 2000; de Medeiros et al., 2010). As a clinician-rated tool, the NPI-C may be a more objective measure of psychotic symptoms in persons with dementia and may support the design of research trials across study design types and settings (de Medeiros et al., 2010).

### 4.4 Defining psychosis in dementia

Psychosis and psychotic symptoms in dementia remain poorly defined in existing literature. Identified tools focused on hallucinations and delusions, however, about 25% of persons with dementia experience “other” psychotic symptoms that are not categorized as hallucinations or delusions (Ropacki and Jeste, 2005). The lack of clarity is reflected in differences in psychosis assessment tools conceptualizing psychosis as total symptom scores or symptom-specific scores (Cohen-Mansfield and Golander, 2011). Studies have found the symptoms of psychosis to be associated with different variables, indicating that there may be value in independently conceptualizing and assessing symptoms (Bassiony et al., 2000).

Commonly used reference standard forms of diagnostic assessment like the DSM are noted to lack symptom specification for psychosis in dementia (Cummings et al., 2020). The IPA diagnostic criteria for psychosis in dementia were created to clearly define specific symptoms of psychosis and consider the timing of symptoms in dementia course (Cummings et al., 2020). The ISTAART-AA criteria were developed to improve the phenotypic classification of psychosis in Alzheimer's Disease, spanning preclinical, prodromal, and dementia states (Fischer et al., 2020). Issues interpreting diagnostic criteria findings as well as issues categorizing psychotic symptoms are thought to impact prevalence estimates of symptoms of psychosis in persons with dementia (Ropacki and Jeste, 2005). As the IPA criteria and complementary ISTAART-AA criteria are specific to psychosis in dementia and Alzheimer's disease, respectively, future diagnostic accuracy studies should evaluate index psychosis tools against the IPA's or ISTAART-AA's reference standard criteria for psychosis assessment to produce the most clinically applicable results.

### 4.5 Why are there so few tools focused on psychosis?

There are seemingly few tools specific to psychosis despite how common psychotic symptoms are for persons living with dementia. A possible explanation may be that psychotic symptoms are more visible to caregivers, either by the person's actions or accounts, thus caregivers or care providers who observe psychotic symptoms may readily report. Alternatively, these symptoms are captured in multiple symptom tools like the NPI. Regardless, having an accurate tool to document symptoms, severity, and track response to therapy would be useful in ensuring measurement-based care.

### 4.6 Limitations

Studies focused primarily on Alzheimer's disease dementia and less so on vascular dementia, dementia with Lewy bodies, or mixed dementia. Study samples ranged in the degree of cognitive function represented most often through MMSE scores. As dementia severity was found to impact correlations between tools for psychosis (Stella et al., 2013), cognitive function, as well as psychotic symptom severity, dementia severity should be considered in future studies. Small sample sizes were a noted limitation in multiple correlation studies. Although a comprehensive search strategy was designed and carried out in multiple electronic databases, it is possible that the search did not identify all studies meeting the inclusion criteria for the primary and secondary objectives. Quantitative analyses of diagnostic accuracy outcomes were not possible given that a single study was identified.

### 4.7 Conclusions

The NPI and CUSPAD were the only psychosis symptom assessment tools evaluated against a reference standard form of psychosis assessment among a variety of index psychosis tools identified. Clinicians may consider the information source, the number of items, and the time required when selecting a psychosis tool. Further research on the comparative utility and diagnostic accuracy is required for all psychosis tools used with persons with dementia.

## Data availability statement

The original contributions presented in the study are included in the article/Supplementary material, further inquiries can be directed to the corresponding author.

## Author contributions

KA: Data curation, Formal analysis, Investigation, Project administration, Writing – original draft. PW: Data curation, Investigation, Writing – original draft. JW: Conceptualization, Writing – review & editing. DS: Conceptualization, Writing – review & editing. ZI: Conceptualization, Writing – review & editing. ZG: Conceptualization, Formal analysis, Supervision, Writing – original draft, Writing – review & editing.
