# Supplementary material for: Psychosis detection in dementia: a systematic review of diagnostic test accuracy studies
Source: Front Dement. 2024 Jun 13;3:1377191. doi: 10.3389/frdem.2024.1377191 (PMC11285683; doi:10.3389/frdem.2024.1377191)
Supplement: Supplementary file 1 [file Table_1.docx]

Supplementary Material

# Supplementary Tables

**Supplementary Table 1.** Database search strategies for the searches completed in MEDLINE, PsycINFO, and Embase on July 20, 2023.

| **Ovid MEDLINE(R) ALL** | |
| --- | --- |
| 1 | exp Dementia/ or Dementia, Multi-Infarct/ |
| 2 | (dement* or alzheimer* or frontotemporal or "lewy bod*" or amentia* or neurocognitiv*).mp. |
| 3 | 1 or 2 |
| 4 | exp "surveys and questionnaires"/ or exp psychological tests/ or exp diagnosis/ or exp "Sensitivity and Specificity"/ or validation study/ |
| 5 | (screen* or tool* or questionnaire* or instrument* or test* or psychometric* or survey* or neuropsych* or inventor*).mp. |
| 6 | ("Psychosis Like Symptoms Semi Structured Interview" or PLIKSi or "Scale for the Assessment of Positive Symptoms Hallucinations and Delusions" or "SAPS-HD" or "SAPS-H+D" or "Burns Symptom Checklist" or "Columbia University Scale for Psychopathology in Alzheimer’s Disease" or CUSPAD or DRP3 or "Dementia Psychosis Scale" or "Behavioral Pathology in Alzheimer’s Disease" or "BEHAVE-AD" or "Neuropsychiatric Inventory" or NPI or "Mild Behavioral Impairment Checklist" or "MBI-C" or "Consortium to Establish a Registry for Alzheimer’s Disease Behavior Rating Scale for Dementia" or "CERAD-BRSD" or "Neurobehavioral Rating Scale" or "Cambridge Examination for Mental Disorders of the Elderly" or CAMDEX).mp. |
| 7 | di.fs. |
| 8 | 4 or 5 or 6 or 7 |
| 9 | (sensitiv* or specificit* or validat* or accurac* or predict* or develop* or "ROC curve" or "receiver operating characteristic").mp. |
| 10 | exp Psychotic Disorders/ or exp Hallucinations/ or Delusions/ |
| 11 | ("psychosis*" or "psychotic*" or "delusion*" or "hallucin*").mp. |
| 12 | 10 or 11 |
| 13 | 3 and 8 and 9 and 12 |
| 14 | exp Schizophrenia/ or exp bipolar disorder/ |
| 15 | (schizophrenia or bipolar).kf,tw. |
| 16 | 14 or 15 |
| 17 | 13 not 16 |
| **APA PsycINFO** | |
| 1 | exp Dementia/ |
| 2 | (dement* or alzheimer* or frontotemporal or "lewy bod*" or amentia* or neurocognitiv*).mp. |
| 3 | 1 or 2 |
| 4 | exp Screening/ or exp Questionnaires/ or exp psychometrics/ or exp diagnosis/ or neuropsychological assessment/ or inventories/ |
| 5 | (screen* or tool* or questionnaire* or instrument* or test* or psychometric* or survey* or neuropsych* or inventor*).mp. |
| 6 | ("Psychosis Like Symptoms Semi Structured Interview" or PLIKSi or "Scale for the Assessment of Positive Symptoms Hallucinations and Delusions" or "SAPS-HD" or "SAPS-H+D" or "Burns Symptom Checklist" or "Columbia University Scale for Psychopathology in Alzheimer’s Disease" or CUSPAD or DRP3 or "Dementia Psychosis Scale" or "Behavioral Pathology in Alzheimer’s Disease" or "BEHAVE-AD" or "Neuropsychiatric Inventory" or NPI or "Mild Behavioral Impairment Checklist" or "MBI-C" or "Consortium to Establish a Registry for Alzheimer’s Disease Behavior Rating Scale for Dementia" or "CERAD-BRSD" or "Neurobehavioral Rating Scale" or "Cambridge Examination for Mental Disorders of the Elderly" or CAMDEX).mp. |
| 7 | 4 or 5 or 6 |
| 8 | (sensitiv* or specificit* or validat* or accurac* or predict* or develop* or "ROC curve" or "receiver operating characteristic").mp. |
| 9 | exp psychosis/ or exp hallucinations/ or delusions/ |
| 10 | ("psychosis*" or "psychotic*" or "delusion*" or "hallucin*").mp. |
| 11 | 9 or 10 |
| 12 | 3 and 7 and 8 and 11 |
| 13 | exp schizophrenia/ or exp bipolar disorder/ |
| 14 | (schizophrenia or bipolar).tw. |
| 15 | 13 or 14 |
| 16 | 12 not 15 |
| **Embase** | |
| 1 | exp dementia/ or Lewy body/ |
| 2 | (dement* or alzheimer* or frontotemporal or "lewy bod*" or amentia* or neurocognitiv*).mp. |
| 3 | 1 or 2 |
| 4 | exp Questionnaires/ or exp screening/ or psychometry/ or "sensitivity and specificity"/ or validation study/ or exp diagnosis/ or neuropsychiatric inventory/ or mental disease assessment/ or clinical assessment tool/ or receiver operating characteristic/ |
| 5 | (screen* or tool* or questionnaire* or instrument* or test* or psychometric* or survey* or neuropsych* or inventor*).mp. |
| 6 | ("Psychosis Like Symptoms Semi Structured Interview" or PLIKSi or "Scale for the Assessment of Positive Symptoms Hallucinations and Delusions" or "SAPS-HD" or "SAPS-H+D" or "Burns Symptom Checklist" or "Columbia University Scale for Psychopathology in Alzheimer’s Disease" or CUSPAD or DRP3 or "Dementia Psychosis Scale" or "Behavioral Pathology in Alzheimer’s Disease" or "BEHAVE-AD" or "Neuropsychiatric Inventory" or NPI or "Mild Behavioral Impairment Checklist" or "MBI-C" or "Consortium to Establish a Registry for Alzheimer’s Disease Behavior Rating Scale for Dementia" or "CERAD-BRSD" or "Neurobehavioral Rating Scale" or "Cambridge Examination for Mental Disorders of the Elderly" or CAMDEX).mp. |
| 7 | di.fs. |
| 8 | 4 or 5 or 6 or 7 |
| 9 | (sensitiv* or specificit* or validat* or accurac* or predict* or develop* or "ROC curve" or "receiver operating characteristic").mp. |
| 10 | exp psychosis/ |
| 11 | ("psychosis*" or "psychotic*" or "delusion*" or "hallucin*").mp. |
| 12 | 10 or 11 |
| 13 | 3 and 8 and 9 and 12 |
| 14 | exp schizophrenia/ or exp bipolar disorder/ |
| 15 | (schizophrenia or bipolar).tw,kw. |
| 16 | 14 or 15 |
| 17 | 13 not 16 |

**Supplementary Table 2.** Grey literature sources searched with total number of results, number of results screened, and references identified for full text screening.

| **Grey Literature Source or Database** | **Search Concepts Used** | **Total number of results** | **Number of results screened** | **Number of references identified for full text screening** |
| --- | --- | --- | --- | --- |
| Centre for Reviews and Dissemination | psychosis AND dementia | 22 | 22 | - |
| ClinicalTrials.gov | psychosis AND dementia | 71 | 71 | - |
| CMA Infobase: Clinical Practice Guidelines | psychosis, dementia | 26 | 26 | - |
| ECRI Guidelines Trust | psychosis AND dementia | 3 | 3 | - |
| European Public Assessment Reports (EPAR) | psychosis AND dementia NOT schizophrenia | 50 | 50 | - |
| Evidence-Based Practice | psychosis AND dementia | 8 | 8 | - |
| Google™ Scholar | psychosis AND dementia; hallucinations AND dementia; delusions AND dementia | 239000; 47000; 54100 | 200; 200; 200 | 17 |
| International Clinical Trials Registry Search Portal | psychosis AND dementia | 14 | 14 | 1 |
| Trip Database - Clinical Search Engine | psychosis AND dementia; hallucinations AND dementia; delusions AND dementia | 4607; 1553; 1055 | 200; 200;200 | 9 |

**Supplementary Table 3.** Study and participant characteristics of articles examining correlations between psychosis tools identified through the systematic review.

| **Author, Year, Country** | **n total, % female** | **Age (mean (SD))** | **Marital status** | **Dementia type** | **MMSE Score (mean (SD))** | **Tools evaluated** | **Prevalence of psychosis reported based on tool** | **Recruitment location & Evaluation location** | **Evaluator & Blinding of evaluator** |
| --- | --- | --- | --- | --- | --- | --- | --- | --- | --- |
| Boada, 2002, Spain^1^ | 60, NR | 76.4 (8.5) | NR | AD | 18.1 (4.7) | NPI-Q-Spanish version [delusions (total, emotional stress), hallucinations (total, emotional stress)]  NPI-Spanish version [delusions (frequency x severity, severity, distress), hallucinations (frequency x severity, severity, distress)] | NR | Cognitive and behavioural disorders unit | NR |
| Boada, 2005, Spain^2^ | 80, 73.8% | 84.0 (9.9) | NR | NR | 11.5 (7.4) | NPI-NH-Spanish version [delusions (total informant rated, total observer rated, occupational disruptiveness)]  NPI-Q-Spanish version [delusions (total, emotional stress)] | NR | Nursing homes | Psychologists; NR |
| Cohen-Mansfield, 2011, Israel^3^ | 74, 76.7% [note: 73 respondents for BEHAVE-AD, 74 for NPI-NH, 37 for BRSD, 36 for CUSPAD] | 85.5 (6.3 SE) | Married: 18.9% | NR | 9.0 (6.8 SE) | BEHAVE-AD [delusions, hallucinations]  NPI-NH [delusions (total, frequency, severity), hallucinations (total, frequency, severity)]  BRSD-SF [delusions (total, frequency), hallucinations (total, frequency)]  CUSPAD [delusions (total, frequency, severity), hallucinations (total, severity)] | BEHAVE-AD [delusions (56.2%), hallucinations (23.3%)]  NPI-NH [delusions (52.7%), hallucinations (24.3%)]  BRSD [delusions (16.2%), hallucinations (8.1%)]  CUSPAD [delusions (75%), hallucinations (19.4%)] | Nursing homes; NR | Research assistant; NR |
| Cummings, 1994, USA^4^ | 40, 45.0% | 75.7 (56-90 range) | NR | AD (50.0%), vascular dementia (22.5%), other (27.5%) | 19.2 (0-29 range) | NPI [delusions (frequency x severity, frequency, severity), hallucinations (frequency x severity, frequency, severity)]  BEHAVE-AD [delusions, hallucinations] | NR | Outpatients of University or Veterans affairs dementia clinic or clinical trials program; NR | NR; NR |
| de Medeiros, 2010, Argentina, Brazil, Canada, France, Greece, Hungary, Italy, USA^5^ | 128, NR | 75.7 (9.0) | Married: 64.3%, widowed: 14.0%, divorced: 7.8%, never married: 14.0% | Probable AD | 17.6 (7.0) | NPI-C (sum of clinician ratings)  NPI [delusions & hallucinations (frequency x severity)]  BPRS [delusions & hallucinations] | NR | Community, nursing homes, assisted living, residential hospitals; NR | Clinicians (includes physicians, research nurses, researchers with master's degree or higher, and clinical social worker); NR |
| Hu, 2023, Canada^6^ | 169, 43.8% (MBI-C-psychosis), 405, 46.2% (NPI-Q-psychosis) | 69.4 (9.4) (MBI-C-psychosis), 68.7 (10.0) (NPI-Q-psychosis) | NR | NR | MOCA: 18.5 (5.7) (MBI-C-psychosis), MOCA: 18.6 (5.5) (NPI-Q-psychosis) | MBI-C [psychosis]  NPI-Q [delusions & hallucinations] | NR | Registry data from cognitive neurosciences clinic; Clinic | NR; NR |
| Silveri, 2004, NR^7^ | 40, NR | 75.74(7.1) | NR | Frontotemporal dementia (27.5%), AD (72.5%) | 18.3 (6.3) | NPI [delusions & hallucinations]  SPAS [psychosis] | NPI-psychosis (60%)  SPAS-psychosis (40%) | NR; NR | Geriatrician; NR |
| Stella, 2013, Brazil^8^ | 156, 73.7% | 76.7 (NR) | NR | Probable AD | 17.2 (NR) | NPI-C [delusions, hallucinations]  BPRS [delusions, hallucinations] | NR | Brazilian centers; NR | Raters; Yes |
| Youn, 2008, South Korea^9^ | 268, 63.8% [note n=24/268 were included in concurrent validation study] | 72.7 (9.7) | NR | Probable AD (56.0%), possible AD (26.9%), mixed (4.9%), vascular dementia (4.5%), other (7.8%) | 14.8 (5.9) | BRSD-Korean [psychosis]  NPI-Korean [delusions (frequency x severity, frequency, severity), hallucinations (frequency x severity, frequency, severity)] | NR | Dementia clinics; NR | NR; NR |
| Ismail, 2013, Canada^10^ | 87, 58.6% | 81.2 (8.5) | NR | Probable AD (78.2%), Possible AD (14.9%), Probable DLB (9.2%), Possible DLB (21.8%) [note 24.1% were both possible AD and DLB] | 9.7 (7.9) [note only n=62/87 participants completed] | E-BEHAVE-AD [psychosis, delusions, hallucinations]  NBRS [psychosis, delusions, hallucinations]  NPI [psychosis, delusions, hallucinations] | NR | Randomized control trial participants recruited from an inpatient unit; NR | NR; NR |

AD: Alzheimer’s Disease; BEHAVE-AD: Behavioral Pathology in Alzheimer’s Disease Rating Scale; BPRS: Brief Psychiatric Rating Scale; BRSD-SF: Behavior Rating Scale for Dementia-Short Form; BRSD-Korean: Behavior Rating Scale for Dementia-Korean Version; CUSPAD: Columbia University Scale for Psychopathology in Alzheimer’s Disease; DLB: Dementia with Lewy Bodies; MBI-C: Mild Behavioral Impairment Checklist; MMSE: Mini-Mental State Examination; MOCA: Montreal Cognitive Assessment; NBRS: Neurobehavioral Rating Scale; NPI: Neuropsychiatric Inventory; NPI-C: Neuropsychiatric Inventory-Clinician Rating Scale; NPI-NH: Neuropsychiatric Inventory-Nursing Home; NPI-Q: Neuropsychiatric Inventory-Questionnaire; NR: Not reported; SE: Standard error; SPAS: Survey Psychiatric Assessment Schedule

**Supplementary Table 4.** Risk of bias of included articles examining correlations between psychosis tools assessed using the COSMIN checklist.

| **Author, Year, Country** | **Box 6. Reliability** | **Box 8. Criterion Validity** | **Box 9. Hypotheses Testing for Construct Validity [9a. Comparison with other outcome measurement instruments (convergent validity)]** |
| --- | --- | --- | --- |
| Boada, 2002, Spain^1^ | Doubtful | - | Adequate |
| Boada, 2005, Spain^2^ | Doubtful | - | Adequate |
| Cohen-Mansfield, 2011, Israel^3^ | Doubtful | - | - |
| Cummings, 1994, USA^4^ | Doubtful | - | - |
| de Medeiros, 2010, Argentina, Brazil, Canada, France, Greece, Hungary, Italy, USA^5^ | Very Good | - | Very Good |
| Hu, 2023, Canada^6^ | Doubtful | - | Adequate |
| Ismail, 2013, Canada^10^ | Very Good | Very Good | Adequate |
| Silveri, 2004, NR^7^ | Doubtful | - | - |
| Stella, 2013, Brazil^8^ | Very Good | - | Very Good |
| Youn, 2008, South Korea^9^ | Very Good | - | Very Good |

Note: Overall quality rating for each study for each box determined by the lowest rating to a signaling question within the box.

**Supplementary Table 5.** Matrix of correlation values between psychosis tools using Spearman’s or Pearson’s correlation coefficients from articles identified through the systematic review.

|  | BEHAVE-AD DL | BEHAVE-AD HL | BPRS DL+HL | BPRS DL | BPRS HL | BRSD-Korean Psychosis | BRSD-SF DL (total score) | BRSD-SF DL (frequency) | BRSD-SF HL (total score) | BRSD-SF HL (frequency) | CUSPAD DL (total score) | CUSPAD DL (frequency) | CUSPAD DL (severity) | CUSPAD HL (total score) | CUSPAD HL (severity) | NPI-Q DL+HL | NPI-Q-Spanish DL | NPI-Q-Spanish DL (emotional stress) | NPI-Q-Spanish HL | NPI Q-Spanish HL (emotional stress) | SPAS Psychosis |
| --- | --- | --- | --- | --- | --- | --- | --- | --- | --- | --- | --- | --- | --- | --- | --- | --- | --- | --- | --- | --- | --- |
| BEHAVE-AD DL |  |  |  |  |  |  | 0.521^§^ (p<0.01)^3^ | 0.456^¶^ (p<0.01)^3^ |  |  | 0.580^§^ (p<0.001)^3^ | 0.682^¶^ (p<0.001)^3^ | 0.557^¶^ (p<0.01)^3^ |  |  |  |  |  |  |  |  |
| BEHAVE-AD HL |  |  |  |  |  |  |  |  | 0.508^§^ (p<0.01)^3^ | 0.409^¶^ (p≤0.05)^3^ |  |  |  | 0.598^§^ (p<0.001)^3^ | 0.645^¶^ (p≤0.001)^3^ |  |  |  |  |  |  |
| MBI-C Psychosis |  |  |  |  |  |  |  |  |  |  |  |  |  |  |  | 0.49^†^ (p<0.00001)^6^ |  |  |  |  |  |
| NPI DL+HL (frequency x severity) |  |  | 0.56^‡^ (95% CI: 0.39-0.69)^5^ |  |  |  |  |  |  |  |  |  |  |  |  |  |  |  |  |  |  |
| NPI DL+HL (presence of sub-questions) |  |  |  |  |  |  |  |  |  |  |  |  |  |  |  |  |  |  |  |  | NR^†^ (NS)^7^ |
| NPI DL (frequency x severity) | 0.76^†^ (p=0.01)^4^ |  |  |  |  |  |  |  |  |  |  |  |  |  |  |  |  |  |  |  |  |
| NPI DL (frequency) | 0.75^†^ (p=0.01)^4^ |  |  |  |  |  |  |  |  |  |  |  |  |  |  |  |  |  |  |  |  |
| NPI DL (severity) | 0.80^†^ (p=0.01)^4^ |  |  |  |  |  |  |  |  |  |  |  |  |  |  |  |  |  |  |  |  |
| NPI HL (frequency x severity) |  | 0.74^†^ (p=0.01)^4^ |  |  |  |  |  |  |  |  |  |  |  |  |  |  |  |  |  |  |  |
| NPI HL (frequency) |  | 0.74^†^ (p=0.01)^4^ |  |  |  |  |  |  |  |  |  |  |  |  |  |  |  |  |  |  |  |
| NPI HL (severity) |  | 0.63^†^ (p=0.01)^4^ |  |  |  |  |  |  |  |  |  |  |  |  |  |  |  |  |  |  |  |
| NPI-Korean DL (frequency x severity) |  |  |  |  |  | 0.393^‡^ (p=0.058)^9^ |  |  |  |  |  |  |  |  |  |  |  |  |  |  |  |
| NPI-Korean DL (frequency) |  |  |  |  |  | 0.334^‡^ (p=0.111)^9^ |  |  |  |  |  |  |  |  |  |  |  |  |  |  |  |
| NPI-Korean DL (severity) |  |  |  |  |  | 0.340^‡^ (p=0.104)^9^ |  |  |  |  |  |  |  |  |  |  |  |  |  |  |  |
| NPI-Korean HL (frequency x severity) |  |  |  |  |  | 0.927^‡^ (p=0.01)^9^ |  |  |  |  |  |  |  |  |  |  |  |  |  |  |  |
| NPI-Korean HL (frequency) |  |  |  |  |  | 0.892^‡^ (p=0.01)^9^ |  |  |  |  |  |  |  |  |  |  |  |  |  |  |  |
| NPI-Korean HL (severity) |  |  |  |  |  | 0.723^‡^ (p=0.01)^9^ |  |  |  |  |  |  |  |  |  |  |  |  |  |  |  |
| NPI-Spanish DL (frequency x severity) |  |  |  |  |  |  |  |  |  |  |  |  |  |  |  |  | 0.929^†^ (p<0.0001)^1^ |  |  |  |  |
| NPI-Spanish DL (severity) |  |  |  |  |  |  |  |  |  |  |  |  |  |  |  |  | 0.925^†^ (p<0.0001)^1^ |  |  |  |  |
| NPI-Spanish DL (distress) |  |  |  |  |  |  |  |  |  |  |  |  |  |  |  |  |  | 0.932^†^ (p<0.0001)^1^ |  |  |  |
| NPI-Spanish HL (frequency x severity) |  |  |  |  |  |  |  |  |  |  |  |  |  |  |  |  |  |  | 0.908^†^ (p<0.0001)^1^ |  |  |
| NPI-Spanish HL (severity) |  |  |  |  |  |  |  |  |  |  |  |  |  |  |  |  |  |  | 0.922^†^ (p<0.0001)^1^ |  |  |
| NPI-Spanish HL (distress) |  |  |  |  |  |  |  |  |  |  |  |  |  |  |  |  |  |  |  | 0.919^†^ (p<0.0001)^1^ |  |
| NPI-C DL+HL |  |  | 0.60^‡^ (95% CI: 0.47- 0.70)^5^ |  |  |  |  |  |  |  |  |  |  |  |  |  |  |  |  |  |  |
| NPI-C DL |  |  |  | 0.713^‡^ (95% CI: 0.626-0.783)^8^ |  |  |  |  |  |  |  |  |  |  |  |  |  |  |  |  |  |
| NPI-C HL |  |  |  |  | 0.432^‡^ (95% CI: 0.295-0.552)^8^ |  |  |  |  |  |  |  |  |  |  |  |  |  |  |  |  |
| NPI-NH DL (total score informant rated) | 0.746^§^ (p<0.001)^3^ |  |  |  |  |  | 0.527^§^ (p<0.01)^3^ |  |  |  | 0.824^§^ (p<0.001)^3^ |  |  |  |  |  |  |  |  |  |  |
| NPI-NH DL (frequency) | 0.739^¶^ (p≤0.001)^3^ |  |  |  |  |  |  | 0.492^¶^ (p≤0.01)^3^ |  |  |  | 0.742^¶^ (p≤0.001)^3^ | 0.745^¶^ (p≤0.001)^3^ |  |  |  |  |  |  |  |  |
| NPI-NH DL (severity) | 0.877^¶^ (p≤0.001)^3^ |  |  |  |  |  |  | 0.509^¶^ (p≤0.01)^3^ |  |  |  | 0.726^¶^ (p≤0.001)^3^ | 0.702^¶^ (p≤0.001)^3^ |  |  |  |  |  |  |  |  |
| NPI-NH-Spanish DL (total score informant rated) |  |  |  |  |  |  |  |  |  |  |  |  |  |  |  |  | 0.372^‡^ (p=0.001)^2^ |  |  |  |  |
| NPI-NH-Spanish DL (total score observer rated) |  |  |  |  |  |  |  |  |  |  |  |  |  |  |  |  | 0.631^‡^ (p≤0.001)^2^ |  |  |  |  |
| NPI-NH-Spanish DL (occupational disruptiveness) |  |  |  |  |  |  |  |  |  |  |  |  |  |  |  |  |  | 0.573^‡^ (p≤0.001)^2^ |  |  |  |
| NPI-NH HL (total score) |  | 0.797^§^ (p<0.001)^3^ |  |  |  |  |  |  | 0.549^§^ (p<0.001)^3^ |  |  |  |  | 0.573^§^ (p<0.001)^3^ |  |  |  |  |  |  |  |
| NPI-NH HL (frequency) |  | 0.772^¶^ (p≤0.001)^3^ |  |  |  |  |  |  |  | 0.442^¶^ (p≤0.01)^3^ |  |  |  |  | 0.545^¶^ (p≤0.01)^3^ |  |  |  |  |  |  |
| NPI-NH HL (severity) |  | 0.798^¶^ (p≤0.001)^3^ |  |  |  |  |  |  |  | 0.425^¶^ (p≤0.01)^3^ |  |  |  |  | 0.58^¶^ (p≤0.001)^3^ |  |  |  |  |  |  |

Note: †: Spearman’s Correlation Coefficient; ‡: Pearson’s Correlation Coefficient; §: Spearman correlations among % of items; ¶: Spearman correlations among severity items; BEHAVE-AD: Behavioral Pathology in Alzheimer’s Disease Rating Scale; BPRS: Brief Psychiatric Rating Scale; BRSD-SF: Behavior Rating Scale for Dementia-Short Form; BRSD-Korean: Behavior Rating Scale for Dementia-Korean Version; CUSPAD: Columbia University Scale for Psychopathology in Alzheimer’s Disease; DL: delusions; HL: hallucinations; MBI-C: Mild Behavioral Impairment Checklist; NPI-C: Neuropsychiatric Inventory-Clinical Rating Scale; NPI-NH: Neuropsychiatric Inventory-Nursing Home; NPI: Neuropsychiatric Inventory; NPI-Q: Neuropsychiatric Inventory-Questionnaire; SPAS: Survey Psychiatric Assessment Schedule; 95%CI: 95% Confidence Interval

# References

1. Boada M, Cejudo JC, Tarraga L, Lopez O, Kaufer D. Neuropsychiatric inventory questionnaire (NPI-Q): Spanish validation of an abridged form of the Neuropsychiatric Inventory (NPI). *Neurologia (Barcelona, Spain)*. 2002;17(6):317-323.

2. Boada M, Tárraga L, Modinos G, López O, Cummings J. Neuropsychiatric inventory-nursing home version (NPI-NH): Spanish validation. *Neurologia (Barcelona, Spain)*. 2005;20(10):665-673.

3. Cohen-Mansfield J, Golander H. The measurement of psychosis in dementia: a comparison of assessment tools. *Alzheimer Disease & Associated Disorders*. 2011;25(2):101-108.

4. Cummings JL, Mega M, Gray K, Rosenberg-Thompson S, Carusi DA, Gornbein J. The Neuropsychiatric Inventory: comprehensive assessment of psychopathology in dementia. *Neurology*. 1994;44(12):2308-2308.

5. de Medeiros K, Robert P, Gauthier S, et al. The Neuropsychiatric Inventory-Clinician rating scale (NPI-C): reliability and validity of a revised assessment of neuropsychiatric symptoms in dementia. *International psychogeriatrics*. 2010;22(6):984-994.

6. Hu S, Patten S, Charlton A, et al. Validating the mild behavioral impairment checklist in a cognitive clinic: comparisons with the neuropsychiatric inventory questionnaire. *Journal of geriatric psychiatry and neurology*. 2023;36(2):107-120.

7. Silveri M, Salvigni B, Jenner C, Colamonico P. Behavior in degenerative dementias: mood disorders, psychotic symptoms and predictive value of neuropsychological deficits. *Archives of gerontology and geriatrics Supplement*. 2004;(9):365-378.

8. Stella F, Forlenza OV, Laks J, et al. The Brazilian version of the Neuropsychiatric Inventory-Clinician rating scale (NPI-C): reliability and validity in dementia. *International psychogeriatrics*. 2013;25(9):1503-1511.

9. Youn JC, Lee DY, Lee JH, et al. Development of a Korean version of the behavior rating scale for dementia (BRSD‐K). *International Journal of Geriatric Psychiatry: A journal of the psychiatry of late life and allied sciences*. 2008;23(7):677-684.

10. Ismail Z, Emeremni CA, Houck PR, et al. A comparison of the E-BEHAVE-AD, NBRS, and NPI in quantifying clinical improvement in the treatment of agitation and psychosis associated with dementia. *The American Journal of Geriatric Psychiatry*. 2013;21(1):78-87.
